# Exploring the Efficacy of Midodrine for Tapering Off Vasopressors

**DOI:** 10.7759/cureus.55192

**Published:** 2024-02-28

**Authors:** Zoraize Moeez Athar, Mahnoor Arshad, Shitij Shrivastava

**Affiliations:** 1 Internal Medicine, BronxCare Health System, New York, USA

**Keywords:** hypotension, septic shock, sepsis, intensive care unit, iv vasopressor, weaning, iv, vasopressor, icu, midodrine

## Abstract

Sepsis and septic shock represent critical conditions, often necessitating vasopressor support in the intensive care unit (ICU). Midodrine, an oral vasopressor, has gathered attention as a potential adjunct to vasopressor therapy, aiming to facilitate weaning and improve clinical outcomes. However, the efficacy of midodrine remains questionable, with conflicting evidence from clinical trials and meta-analyses. This article provides a comprehensive review of the literature on midodrine's role in ICU settings by gathering evidence from multicenter trials, retrospective studies, and meta-analyses. While some studies suggest a limited benefit of midodrine in expediting vasopressor weaning and reducing ICU/hospital stays, others report potential advantages, particularly in reducing mortality rates among septic shock patients. Ongoing efforts aim to address knowledge gaps surrounding midodrine's efficacy and safety.

## Introduction and background

Sepsis is a serious condition that is described as a dysregulated systemic response to infection leading to organ damage and septic shock. According to the Third International Consensus Definitions for Sepsis and Septic Shock, septic shock is defined as a subset of sepsis in which underlying circulatory and cellular/metabolic abnormalities are profound enough to substantially increase mortality [1]. It may lead to dangerously low blood pressure requiring the administration of vasopressors. Circulatory shock affects about one-third of patients who get admitted to the intensive care unit (ICU). About 2-3% of patients in ICU suffer from septic shock, and the mortality rate is reported to be as high as 50% [2]. Such patients often need vasopressors and inotropes, which are a class of drugs that induce vasoconstriction and elevate the mean arterial pressure. They are very potent pharmacological agents. There are different kinds of vasopressors such as norepinephrine, vasopressin, epinephrine, phenylephrine, and dobutamine to name a few, each having a different mechanism of action. Midodrine is an oral vasopressor that was patented in 1965 in Austria [3,4] and was first reported in 1970 as a novel orally administered peripherally acting alpha receptor agonist with good enteral absorption [3,5]. It increases blood pressure by raising systemic vascular resistance. Multiple retrospective, prospective, and randomized controlled clinical trials have demonstrated the use of midodrine as a weaning agent in ICU [6-9]. It was first approved by the Food and Drug Administration (FDA) in 1996 through an accelerated program for the treatment of dysautonomia and orthostatic hypotension. In addition to its primary use, midodrine has been explored for its potential role in weaning patients off vasopressor support in intensive care settings. It is still not FDA-approved for weaning patients from vasopressor support. Other drugs such as droxidopa and pseudoephedrine are available as well but all are off-label uses. Contrasting and conflicting evidence has been reported in the use of midodrine for weaning patients off low-dose vasopressors [6,10]. One study reported that midodrine has no effect on the time to vasopressor discontinuation and does not accelerate liberation from vasopressors [6], while another study [10] reported that the use of midodrine significantly lowered intravenous (IV) vasopressor duration. Due to such inconsistent results and divergent findings, further studies are required. This article will focus on whether midodrine is effective as an adjunct to low vasopressor therapy to facilitate the tapering of vasopressors and reduce the time on vasopressors, via a traditional review of literature.

## Review

Increasing trends in the use of midodrine as a vasopressor-sparing agent

Despite limited evidence of safety in the ICU setting, midodrine has been increasingly used as a vasopressor-sparing agent. A single-center retrospective case series from January 2011 to October 2016 at Mayo Clinic, Rochester, studied the current practices regarding midodrine in the ICU [8]. During the study, 1,119 patients were given midodrine, predominantly in surgical and medical ICUs. After initiation, there was a notable reduction in patients needing vasopressors, alongside a decrease in cumulative vasopressor doses among those still requiring them. Patients not on vasopressors experienced a rise in median MAP. The most frequent side effect observed was asymptomatic bradycardia, occurring in 172 patients, with a median decrease in heart rate of 39 beats per minute [8]. The findings indicate a growing trend of utilizing midodrine in ICU settings to augment MAP and aid in the tapering from vasopressors. However, prospective trials are needed to better determine the optimal timing, effectiveness, safety, and cost-effectiveness of midodrine administration among ICU patients.

Evidence showing no significant benefit of midodrine

The MIDAS trial was a multicenter, randomized controlled trial (RCT) conducted in three tertiary referral hospitals in the United States and Australia that involved adult patients experiencing hypotension requiring a single-agent IV vasopressor for at least 24 hours. Participants were randomly assigned to receive either oral midodrine (20 mg) or a placebo every eight hours alongside standard care until discontinuation of IV vasopressors, discharge from the ICU, or occurrence of adverse events. The primary outcome measured was the time taken for vasopressor discontinuation. Secondary outcomes included time to readiness for ICU discharge, length of stay (LOS) in the ICU and hospital, and ICU readmission rates. Between October 2012 and June 2019, a total of 136 participants were randomized for the study. Out of these, 132 received their assigned intervention and were included in the analysis following a modified intention-to-treat approach. The time taken for vasopressor discontinuation did not significantly differ between the midodrine and placebo groups (median 23.5 hours vs 22.5 hours, p=0.62). Additionally, no significant variances were observed in secondary endpoints. However, bradycardia occurred more frequently following midodrine administration compared to the placebo group (five vs zero patients, p=0.02). The trial effectively concluded that midodrine did not expedite weaning from vasopressors and its utilization was not effective in the treatment of hypotension in critically ill patients [6].

The MAVERIC study was a pilot study that was conducted as an open-label, RCT involving patients from two tertiary ICUs who were on low-dose IV vasopressor therapy for more than 24 hours. Patients were randomly assigned to receive either adjunctive midodrine (10 mg every eight hours) or usual care. The primary efficacy outcome measured was the time taken for IV vasopressor therapy to be stopped. Secondary outcomes included protocol compliance and LOS in the ICU and hospital. Over a span of 22 months, 381 patients were screened, and 62 were enrolled (32 in the midodrine group, 30 in the usual care group). The median time for cessation of vasopressor infusion was 16.5 hours for the midodrine group compared to 19 hours for the usual care group (p=0.22). ICU stay was 50 hours for midodrine vs. 59 hours for usual care (p=0.14), and hospital LOS did not differ significantly either. Protocol compliance was high at 96.9%. One patient stopped midodrine early due to symptomatic bradycardia. While adjunctive midodrine therapy demonstrated acceptable compliance, duration, and safety, there was no evidence of physiological or clinical efficacy at the chosen dose [11].

Another clinical review by Smith et al. summarized five studies with a total of over 1,000 patients conducted between 2011 and 2021. Observational studies suggested that administering midodrine led to quicker liberation from IV vasopressor therapy and shorter ICU stays in patients recovering from vasodilatory shock. However, these results were not confirmed in a prospective, multicenter, RCT, hence, failing to prove midodrine’s effectiveness as a potential weaning agent to vasopressor use [12].

Another single-center, case-control study was conducted between 2012 and 2020 at Sir Charles Gairdner Hospital in Perth, Australia, parallel to the MIDAS study. Patients identified as cases by treating intensivists received 20 mg of oral midodrine every eight hours, while controls were given a placebo. Data on vasopressor infusion duration and dose, hemodynamics, and adverse events were collected. Between 2012 and 2019, 42 controls and 19 cases were recruited. Cases, who received midodrine, had a median vasopressor infusion duration of 94 hours compared to 29.3 hours for controls, indicating prolonged vasopressor dependence among cases. However, midodrine use did not result in faster weaning from IV vasopressors (26 hours vs. 24 hours for controls, p=0.51) or shorter ICU or hospital stays after adjusting for confounders. Midodrine did not significantly affect the mean heart rate but was linked to bradycardia. This case-control study suggests that midodrine has limited efficacy in expediting weaning from vasopressor infusions in patients who have already received prolonged courses of these infusions [13].

A meta-analysis by Hammond et al. inclusive of three studies comprising of 2533 patients was conducted. Patients who received midodrine in addition to IV vasopressor therapy showed no significant difference in ICU LOS (mean difference of 1.38 days, 95% confidence interval=-3.48-6.23), or hospital LOS (mean difference of 4.37 days, 95% confidence interval=-3.45-12.19) compared to those receiving IV vasopressor therapy alone. The duration of IV vasopressor therapy post-midodrine initiation also showed no significant difference (mean difference of 7.28 days, 95% confidence interval=-0.86-15.41). Mortality rates were similar between the two groups (odds ratio: 0.74, 95% confidence interval: 0.44-1.27). Reporting biases were minimal, but study heterogeneity and limitations in availability influenced the results. The study concluded that midodrine had no effect on ICU or hospital LOS [14].

Another meta-analysis by Hamed et al. included four RCTs with a total of 314 patients. No significant difference was analyzed in the total duration of vasopressor use in the midodrine and control group (mean difference of -0.53, p=0.22). Additionally, no difference was noted in the time to vasopressor cessation (mean difference of -0.05, p=0.09) and ICU/hospital LOS. On the contrary, the midodrine group was associated with a higher risk of bradycardia [15].

Evidence showing midodrine might be useful 

A study randomized 60 patients who required IV norepinephrine use for more than 24 hours. The two cohorts included a group that added low-dose midodrine along with IV norepinephrine, while the other group continued with norepinephrine only. Results revealed that the midodrine group had a significantly shorter median duration of IV norepinephrine (four days vs. six days) and a shorter norepinephrine weaning time (26 hours vs. 78.5 hours, p=<0.001) compared to the norepinephrine-only group. Moreover, the midodrine group exhibited a lower mortality rate (43.3% vs. 73.3%, p=0.018) while the LOS in the hospital was comparable between the two groups. Importantly, the midodrine group demonstrated cost-saving results compared to the norepinephrine-only group [10].

Another study conducted a retrospective comparison of adult ICU patients admitted to the institution over a period of five years. The analysis included 188 patients, with 94 receiving midodrine and 94 serving as controls. Patients discontinued IV vasopressors a median of 1.2 days after midodrine initiation, with 96% remaining off IV vasopressors after midodrine treatment. ICU discharge occurred sooner after IV vasopressor discontinuation in the midodrine group (p=0.01), but hospital LOS was longer in these patients(p=<0.01). There were no significant differences in ICU LOS or readmissions [16].

Whitson et al. performed a study in a medical ICU focusing on patients diagnosed with septic shock requiring at least 24 hours of IV vasopressors, who exhibited clinical stability with stable or decreasing doses of IV vasopressors. Two groups were compared: patients who received IV vasopressors alone and those who received IV vasopressors along with adjunctive midodrine. Among the 275 study patients, 140 received IV vasopressors only, while 135 received midodrine in addition to IV vasopressors. Demographic characteristics such as age, sex, and acute physiology and chronic health evaluation 4 score did not differ significantly between the groups. The mean duration of IV vasopressor use was significantly shorter in the IV vasopressor with midodrine group compared to the IV vasopressor-only group (2.9 days vs. 3.8 days, respectively; p<0.001). Reinstatement of IV vasopressors after discontinuation was less common in the IV vasopressor with midodrine group (5.2%) compared to the IV vasopressor-only group (15%) (p=0.007). ICU LOS was also shorter in the IV vasopressor with midodrine group compared to the IV vasopressor-only group (7.5 days vs. 9.4 days, respectively; p=.017). No complications associated with midodrine use were reported except for transient bradycardia in one patient, which resolved upon discontinuation of midodrine [17].

Another prospective study involved 60 patients admitted to the adult ICU with septic shock. The patients were randomly assigned to one of two groups (midodrine vs standard therapy), each consisting of 30 patients. The study found that the administration of midodrine had a highly significant effect on the ICU LOS (p=<0.001) and ICU mortality rates (p=<0.001). Specifically, the ICU LOS was significantly shorter in the midodrine group compared to the control group (6.2 vs. 8.3 days), and the ICU survival rate was markedly higher in the midodrine group compared to the control group (96.7% vs 60%). The study suggests that several confounding factors, such as provider confidence in patient stability, bed availability in other units, or continued need for ICU monitoring for other reasons, might have influenced these outcomes [18].

A meta-analysis was conducted by He et al. which included six studies. The analysis found that adding midodrine to patients with septic shock was associated with a reduction in both hospital mortality (risk ratio: 0.76, p=0.05) and ICU mortality (risk ratio: 0.59, p=0.008). However, there were no significant differences observed in the duration of IV vasopressors, IV vasopressor reinstitution, ICU LOS, and hospital LOS between the midodrine group and the IV vasopressor alone group. The study concludes that the additional use of midodrine might reduce hospital and ICU mortality in patients with septic shock, but more high-quality RCTs are needed to confirm this conclusion [19].

Future trials

The LIBERATE study is a planned multicenter trial that will involve adult critically ill patients (age≥18 years) who are receiving stable or decreasing doses of IV vasopressors. Participants will be randomly assigned to receive either midodrine 10 mg administered enterally every eight hours or placebo until 24 hours after discontinuation of IV vasopressors. The primary outcome measure will be the LOS in the ICU. Secondary outcomes will include all-cause mortality at 90 days, hospital LOS, duration of IV vasopressor support, re-initiation of IV vasopressors, rates of ICU readmission, and occurrence of adverse events [20].

Discussion

The exploration of midodrine's efficacy for facilitating the tapering of vasopressors in ICU patients presents an interesting situation defined by conflicting evidence. Despite its increasing utilization as a vasopressor-sparing agent in critical care settings, the effectiveness of midodrine remains a subject of debate.

Several studies, including the MIDAS trial and the MAVERIC study, have yielded evidence suggesting a limited benefit from midodrine therapy in expediting the weaning of patients off vasopressors. The MIDAS trial, a multicenter RCT, and the MAVERIC study, an open-label RCT, both failed to demonstrate a significant reduction in the time to vasopressor discontinuation or ICU/hospital LOS with midodrine administration [6,11]. Moreover, meta-analyses by Hammond et al. and Hamed et al. incorporating multiple studies have consistently shown no substantial benefits of midodrine in terms of ICU or hospital LOS, duration of IV vasopressor therapy, or mortality rates [14,15].

However, amidst the prevailing uncertainty, certain studies have reported potential advantages associated with midodrine therapy. Some studies have suggested that midodrine administration may lead to quicker liberation from IV vasopressor therapy and shorter ICU stays in patients recovering from vasodilatory shock [10,16,18]. Furthermore, studies such as the one conducted by Whitson et al. and a meta-analysis by He et al. incorporating six studies reported a reduction in hospital and ICU mortality rates among septic shock patients receiving midodrine adjunctively [17,19].

The conflicting evidence highlights the need for further research to define the role of midodrine in vasopressor weaning and septic shock management. The ongoing LIBERATE study represents a significant step towards addressing this gap in knowledge. By rigorously evaluating the efficacy, safety profile, and cost-effectiveness of midodrine in critically ill patients, the LIBERATE trial aims to provide valuable insights that can inform clinical practice and guide treatment decisions in the ICU [20].

## Conclusions

Midodrine continues to be used based on clinician preference. Due to its tolerable safety profile and pharmacological target receptors, it can be said to be an appropriate choice. It is important to conduct well-designed, multicenter RCTs to clarify the impact of midodrine on clinical outcomes, identify patient subgroups that may benefit most from its use, and establish optimal dosing regimens. Only through comprehensive studies, we can unlock the true potential of midodrine as a therapeutic adjunct in the management of septic shock and vasopressor weaning in critically ill patients.
